# Personalized radiation dosimetry for PRRT—how many scans are really required?

**DOI:** 10.1186/s40658-020-00293-z

**Published:** 2020-05-11

**Authors:** Nanette Freedman, Mattias Sandström, Jonathan Kuten, Natan Shtraus, Inna Ospovat, Albert Schlocker, Einat Even-Sapir

**Affiliations:** 1 Institute of Nuclear Medicine, Tel Aviv Sourasky Medical Center, 6 Weizman Street, 64239 Tel Aviv, Israel; 2 Section of Nuclear Medicine and PET, Department of Surgical Sciences, Uppsala University, Uppsala, Sweden; 3 Institute of Radiotherapy, Tel Aviv Sourasky Medical Center, Tel Aviv, Israel; 4 Sackler School of Medicine, Tel Aviv University, Tel Aviv, Israel

**Keywords:** Method dependence, Dosimetry, ^177^Lu-DOTATATE, Neuroendocrine tumors

## Abstract

**Purpose:**

Over recent years, peptide receptor radiotherapy (PRRT) has been recognized as an effective treatment for patients with metastatic neuroendocrine tumors (NETs). Personalized dosimetry can contribute to improve the outcome of peptide receptor radiotherapy (PRRT) in patients with metastatic NETs. Dosimetry can aid treatment planning, ensuring that absorbed dose to vulnerable normal organs (kidneys and bone marrow) does not exceed safe limits over serial treatments, and that absorbed dose to tumor is sufficient. Absorbed dose is estimated from a series of post-treatment SPECT/CT images. Total self-dose is proportional to the integral under the time activity concentration curve (TACC). Method dependence of image-based absorbed dose calculations has been previously investigated, and we set out here to extend previous work by examining implications of number of data points in the TACC and the numerical integration methods used in estimating absorbed dose.

**Methods:**

In this retrospective study, absorbed dose estimates and effective half-lives were calculated by fitting curves to TACCs for normal organs and tumors in 30 consecutive patients who underwent a series of 4 post-treatment SPECT/CT scans at 4 h, 24 h, 4–5 days, and 1 week following ^177^Lu-DOTATATE PRRT. We examined the effects of including only 2 or 3 rather than all 4 data points in the TACC, and the effect of numerical integration method (mono-exponential alone or in combination with trapezoidal rule) on the absorbed dose and half-life estimates. Our current method is the combination of trapezoidal rule over the first 24 h, with mono-exponential fit thereafter extrapolated to infinity. The other methods were compared to this current method.

**Results:**

Differences in absorbed dose and effective half-life between the current method and estimates based only on the second, third, and fourth scans were very small (mean differences < 2.5%), whereas differences between the current method and 4-point mono-exponential fit were higher (mean differences < 5%) with a larger range. It appears that in a 4-point mono-exponential fit the early (4 h) time point may skew results, causing some large errors. Differences between the current method and values based on only 2 time points were relatively small (mean differences < 3.5%) when the 24 h and 1 week scans were used, but when the 24 h and 4–5 days scans, or the 4–5 days and 1 week scans were used, differences were greater.

**Conclusion:**

This study indicates that for ^177^Lu-DOTATATE PRRT, accurate estimates of absorbed dose for organs and tumors may be estimated from scans at 24 h, 72 h, and 1 week post-treatment without an earlier scan. It may even be possible to cut out the 72 h scan, though the uncertainty increases. However, further work on more patients is required to validate this.

## Introduction

In recent years, peptide receptor radiotherapy (PRRT) using ^177^Lu-DOTATATE has been demonstrated to be effective for treatment of patients with metastatic neuroendocrine tumors (NETs) [1–3]. ^177^Lu-DOTATATE binds to somatostatin receptors present in NETs. ^177^Lu decays mainly in the form of β radiation, with a small part of γ radiation. The β radiation delivers effective radiotherapy to the immediately surrounding tissue, with little collateral damage to other organs. The γ radiation (main energy 208 keV) permits post-treatment imaging that can be used to confirm that the radiopharmaceutical has reached its intended targets (primary tumor and/or metastases) and also for personalized dosimetry.

Personalized dosimetry calculations based on post-therapy SPECT images yield estimates of absorbed dose to tumors and normal tissues [4]. These absorbed dose estimates contribute to the planning of ongoing serial treatments, to avoid exceeding the absorbed dose threshold for vulnerable normal organs and to ensure sufficient absorbed dose to tumor, in the light of evidence of an absorbed dose-response curve, at least for pancreatic neuroendocrine tumors [5].

Absorbed dose to solid organs is essentially self-dose due to quite high activity within the organ, with a negligible contribution from cross-dose due to activity in other organs [6]. Total absorbed dose due to time-integrated activity concentration is given by the integral under the TACC multiplied with the appropriate factor (ACDF). While it is not practical to acquire images at more than 3–4 time points, it is also not possible to calculate an exact integral based on so few time points, and approximations must be made.

Others have addressed issues of reducing the number of post-treatment scans, even suggesting methods based on a single scan [7–11], but so far, the dependence on time points used for the suggested methods for dosimetry based on fewer scans has mostly been widely tested only for kidney dosimetry and not generally validated for normal organs and tumors. Furthermore, validation was not always performed with late time points (1 week) included in the reference method, as recommended by MIRD [12] to avoid substantial extrapolations in the calculation of absorbed dose. Our initial data demonstrated variability of the time course of ^177^Lu-DOTATATE in normal organs and to an even greater extent in tumors beyond that previously reported. This prompted us to investigate the time points as well as the numerical integration methods used to calculate absorbed dose and effective half-life, in the context of the observed diversity of TACCs including a late (1 week) time point.

This retrospective study examined TACCs for normal organs and tumors from patients receiving PRRT at our institution. To provide accurate estimates of absorbed dose to normal organs and tumors, while maintaining careful use of resources and not acquiring more images than necessary, we used the activity concentration data acquired in our current treatment protocol to investigate the following methodological questions: (i) impact of numerical integration method (single exponential fit, trapezoidal rule, or combination of these 2 methods) on estimates of absorbed dose and effective half-life; (ii) are 4 time points really required to obtain accurate dose estimates, or will 3 or even 2 points suffice, and if so which points can be omitted without compromising the results?

## Methods

### Patients and treatment protocol

Anonymized data from the first 30 patients who received ^177^Lu-DOTATATE PRRT and completed post-therapy imaging at our institution (Tel Aviv Sourasky Medical Center, Tel Aviv, Israel) were analyzed. Treatment protocols were as described in the literature [2, 13]. In brief, patients received approximately 7.4 GBq ^177^Lu-DOTATATE in each treatment, diluted in saline and administered iv over a period of 30 min and also received an iv infusion of amino acids (l-lysine and l-arginine) as specified by the FDA [14], commencing 30 min before the start of the PRRT and continuing at least 3 h after, to reduce absorbed dose to kidneys.

Post-treatment imaging included SPECT/CT images approximately 4 h, 24 h, 4–5 days, and 6–7 days after initiation of treatment. All images were acquired on a GE Optima NM/CT 640. SPECT images were acquired using a Medium Energy General Purpose (MEGP) collimator, acquiring 120 frames with a 30-s exposure time per frame (total acquisition time 30 min). Coregistered CT was acquired on the four-slice CT (140 kVp, 3.0 mA and half rotation) and used to generate an attenuation map. For SPECT reconstruction, the ordered subsets expectation maximisation (OSEM) algorithm included in the Xeleris 3.0 workstation (International General Electric, General Electric Medical Systems, Haifa, Israel) was used with default settings (iterative reconstruction with eight subsets and four iterations followed by a Hann filtering with a cut-off of 0.85). The images were attenuation corrected with the CT-created attenuation map.

IRB approval was given for this retrospective anonymized study.

### Image analysis

Images were analyzed using the GE Dosimetry Toolkit to co-register serial SPECT/CT images, define VOIs for normal organs (left and right kidneys, spleen, liver) and for 3–4 tumor foci on all images, and calculate the corresponding TACCs. VOIs were defined on the SPECT images, with assistance of co-registered CT. VOIs were transferred automatically to all co-registered images in the series, with the option of manual user adjustment in case of need, as occurred particularly for tumor foci situated in soft tissue. In patients with tumor involvement in the liver, definition of VOIs including only normal liver was often challenging and limited to small sections of the liver.

### Dosimetry

To permit conversion of data in counts to activity concentration the SPECT system was calibrated using phantom acquisitions including a known amount of activity in a known volume [4, 13] chosen to correspond to volumes typically used for VOIs defined for organs and tumors in the dosimetry.

Absorbed dose for an organ or tumor is calculated by multiplication of time-integrated activity concentration with the appropriate ACDF [4, 15, 16].

To calculate the time-integrated activity concentration, the TACC is typically described as mono- or bi-exponential washout of the injected activity from organs, and the integral is calculated directly from the parameters of the exponential(s). Bi-exponential washout includes a brief rapidly descending first phase, followed by a slower washout from there on. Frequently, the fast component of the washout is so brief that the data will not support a bi-exponential fit, and fitting the TACC to a single exponential yields a good approximation, and is therefore widely used for dosimetry, making it possible to base dose calculation on fewer time points. It was our original intention to use this option. However, with the first time point only 4 h post-injection, we often saw evidence of the initial fast decreasing exponential component, as reported by Delker et al. [17] for the case of kidney dosimetry, or, more surprisingly, an initial increase in activity apparently extending beyond 4 h post-injection before the curve started to decrease in mono-exponential washout.

**Fig. 1:**
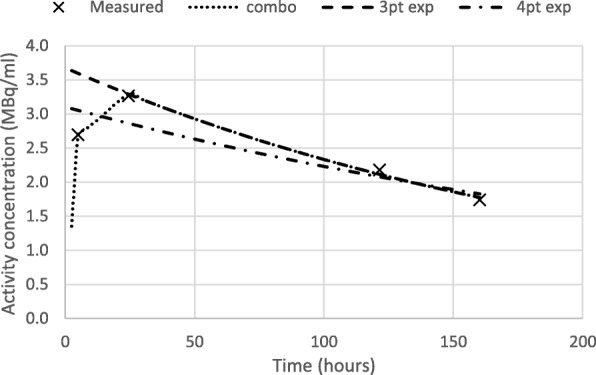
Example illustrating the different extrapolations of the TACC used for calculation of absorbed dose and effective half-life. The trapezoidal rule from injection to 24 h is used in the “original” combined method, and curve fits to a 3- or 4-point mono-exponentials are shown

As a result of this variability in initial behavior, inclusion of an early time point in a mono-exponential fit to estimate area under the curve may not always be accurate. Examples of this are shown in Fig. 1. To avoid rigid assumptions of the behavior of the curve during this initial time, we considered a combined method, estimating the integral under the curve as the sum of (i) trapezoidal integration from *t* = 0 up to the second time point (approximately 24 h) and (ii) the area under a mono-exponential fitted to time points 2, 3, and 4 (from 24 h on), extrapolated from injection out to infinity. We refer to this method as the “combined trapezoidal/mono-exponential” absorbed dose estimate. To decide which method to use as our “original method,” we compared the combined method to the option simply to use all 4 time points for a mono-exponential fit to all 4 time points. While mono-exponential fit of 4 standard time points also yielded a good fit for most normal organs and tumors, poor fit (as judged visually—for examples, see Fig. 1) as well as referring to the coefficient of determination *R*^2^ (see the “Statistics” section below) was more frequent when all four time points were fitted to a mono-exponential than in the combined method where the early time point was not included in the mono-exponential fit. We therefore use the “combined trapezoidal/mono-exponential” method for estimating dose and half-life from 4 time points as our reference method in this study and refer to it as our “original method.” Note that in this “original method,” points from 24 h on are fitted to a mono-exponential, and we considered the half-life based on this fit to characterize the long-term washout of ^177^Lu-DOTATATE. We compared this “original method” to the mono-exponential fit based on all 4 time points and to the alternative options of estimating dose using mono-exponential fit to 3 and 2 time point TACCs obtained by omitting time points.

### Statistics

Time activity concentration curves (TACC’s) of activity concentration, C, (MBq/ml) measured at 2, 3, or 4 data points were fitted to a mono-exponential:

1 $$ {C}_t={C}_0\times {e}^{-\ln (2)t/{t}_{eff}} $$

where *C*_t_ is the activity concentration at time t, *C*_0_ is the activity concentration at time zero obtained from the curve fit, and *t*_eff_ is the effective half-life in therapy obtained from the curve fit.

Curve fitting was performed using the least squares method to obtain a fit to the activity concentrations, and the coefficient of determination *R*^2^ was used as an indicator goodness of fit, with *R*^2^ > 0.95 considered to be a good fit.

Note that both *C*_0_ and *t*_eff_, representing initial uptake and half-life for washout, are crucial parameters in determining absorbed dose.

Bland-Altman plots, plotting percent differences in absorbed dose (or half-life) versus the mean values estimated using the original method and the method of choice, were used to compare the methods. To summarize the results of these comparisons, we also calculated the mean percent differences between each method and the “original method” and the 95% confidence interval, indicating the uncertainty in these mean differences. To demonstrate the range of the differences between each method and the “original method” that may be expected over individual patients, we also calculated the 95% percentile interval, taking into account both within and between patient variation for tumor foci and the kidneys and the proportion of tumors and organs with absolute percent difference > 10%.

## Results

Four SPECT/CT scans were acquired 5.0 ± 0.7 (mean ± standard deviation), 23.4 ± 1.0, 112.9 ± 13.0, and 165.1 ± 10.0 h post-treatment. VOIs were defined for normal organs and tumor foci for each patient, and time activity concentration curves (TACCs) were generated.

**Fig. 2:**
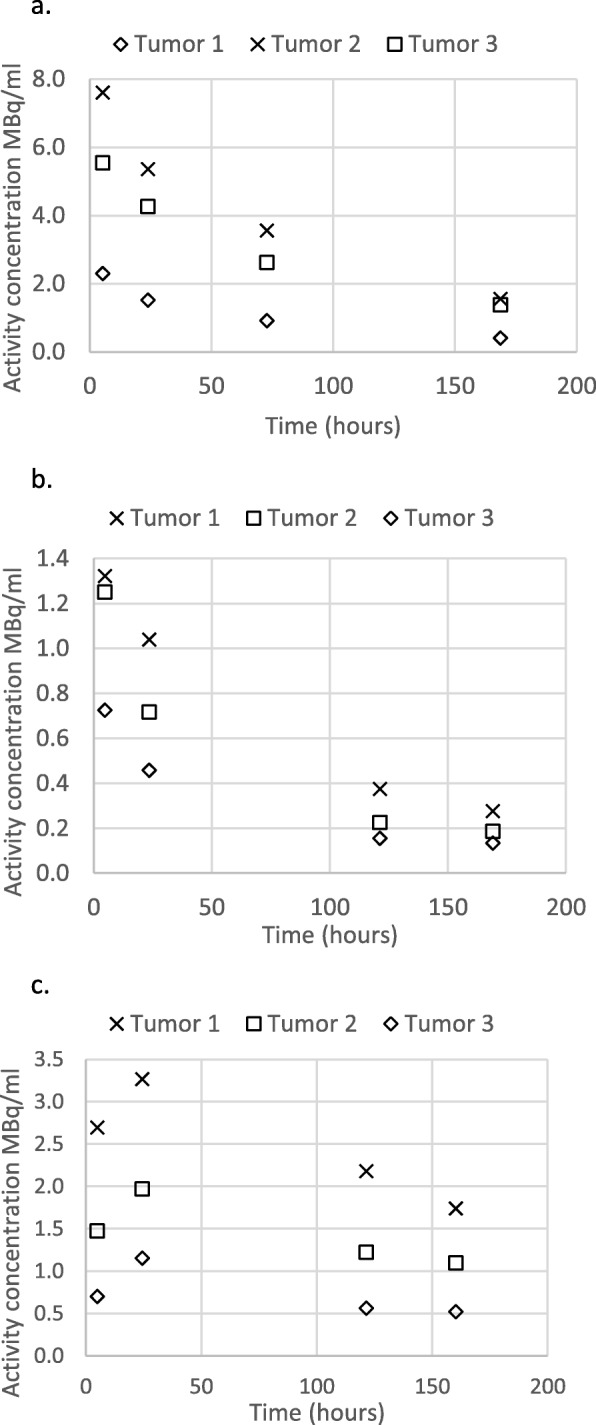
Examples of time activity concentration curves for tumors, showing **a** mono-exponential decrease; **b** bi-exponential, with initial steeper decrease over first 24 h approximately, followed by mono-exponential decrease; **c** initial increase over first 24 h approximately, followed by mono-exponential decrease. Note that in all these graphs, tumors are labelled 1, 2, and 3 only to distinguish the curves from each other, and there is no significance to the numbering

Visual inspection of the TACCs for normal organs and tumors confirmed the variability described in the “Methods” section. Specifically, while many TACCs followed a single exponential overall (Fig. 2a), in the initial 4–24 hours post treatment, a second more steeply decreasing exponential component (Fig. 2b) was evident in at least one TACC in 22 of 30 patients, or alternatively, an initial increase preceded the subsequent exponential decrease (Fig. 2c) in at least one TACC in 15 of 30 patients. TACCs obviously diverging from single exponential washout were, as suspected, seen most frequently in tumors but also in some normal organs. These visual observations together with the corresponding exponential fits with *R*^2^ > 0.95 confirmed our decision to estimate absorbed dose using the combined trapezoidal/mono-exponential method described above (referred to here as the “original method”).

### Absorbed dose and effective half-life for normal organs and tumors

**Fig. 3:**
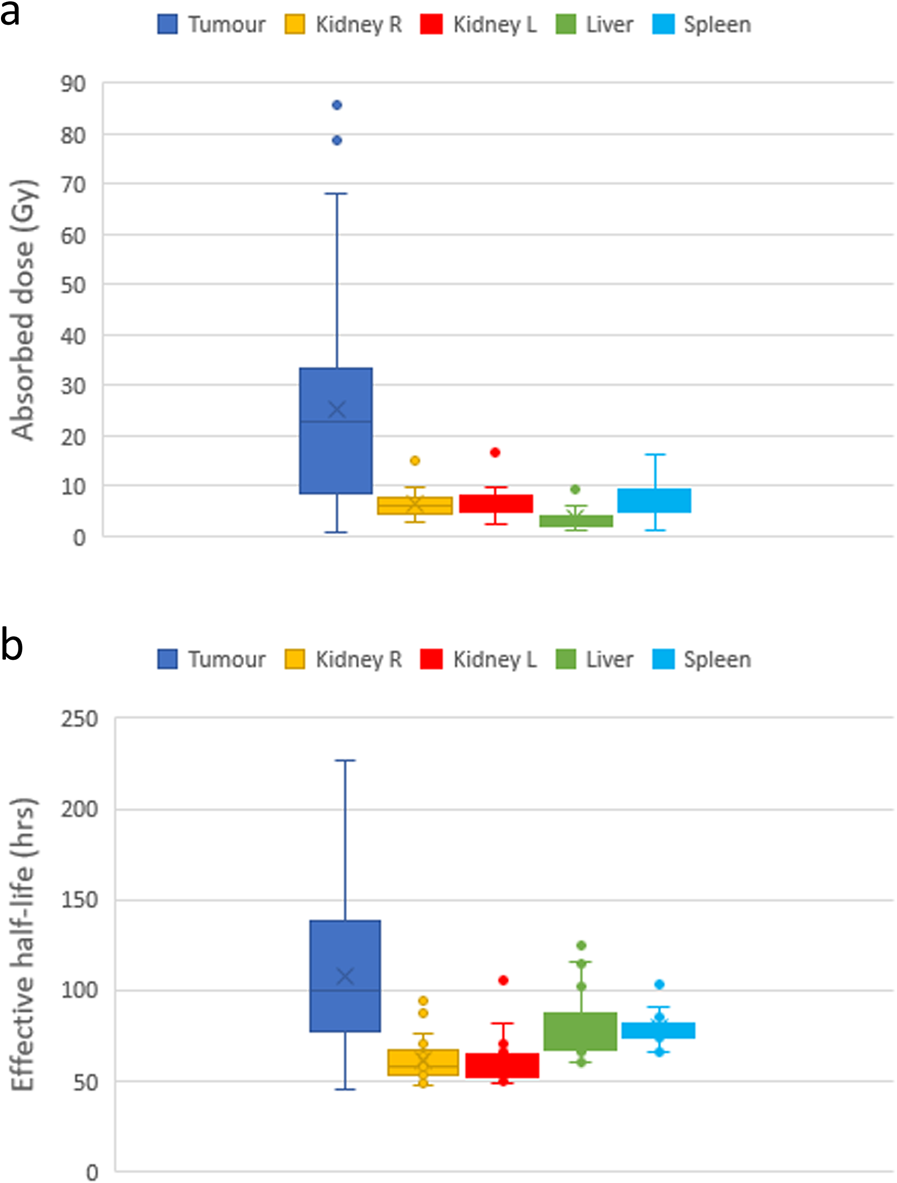
Box-whisker plots for absorbed dose (**a**) and effective half-life (**b**) for the tumors, kidneys, liver, and spleen.

Absorbed dose and effective half-life estimates are shown in box-whisker plots in Fig. 3a and b, respectively. These data indicate both higher absorbed dose and longer effective half-life for tumors as compared to normal organs, as expected. The mono-exponential fit of 3 standard time points (24 hr, 4–5 days, 6–7 days) yielded a good fit (*R*^2^ > 0.95) for all normal organs (R and L kidneys, liver, spleen) in 25/30 patients and for all tumors in 21 patients; mono-exponential fit was somewhat less good (0.90 > *R*^2^ > 0.95) for the remaining organs and tumors and failed for all three tumors in one patient. Data for these 3 tumors were not included in the comparisons between methods reported below.

### Comparison of 4-time point mono-exponential fit from 0 to infinity vs “original method”

**Fig. 4:**
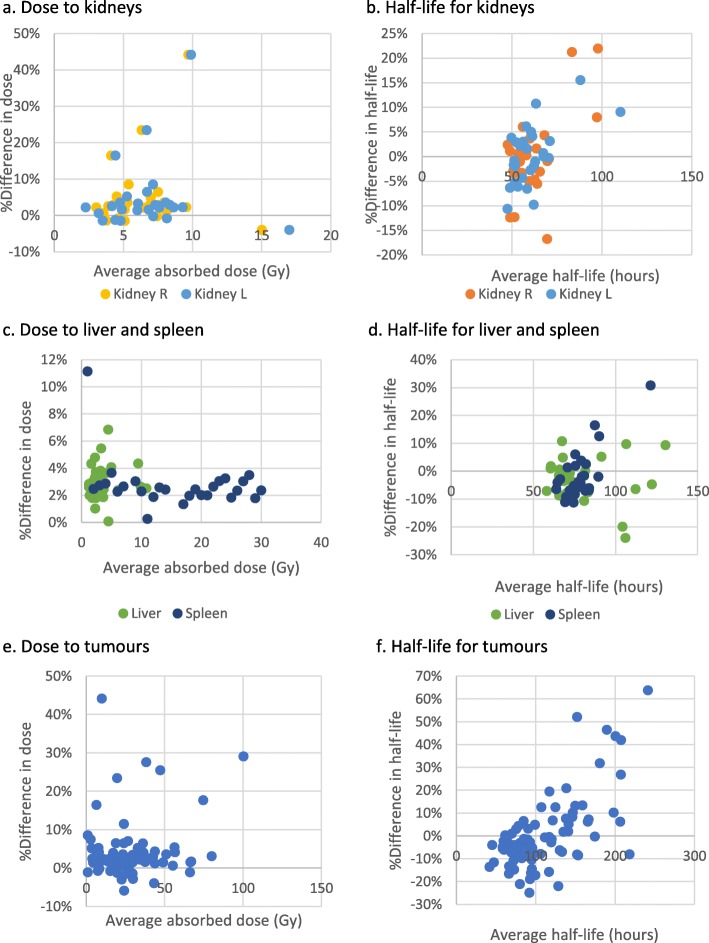
Bland-Altman plots showing % difference between 4-point mono-exponential fit and “original method” (combined trapezoidal/3-point-mono-exponential fit) for absorbed dose (**a**) and effective half-life (**b**) in the kidneys, liver and spleen (**c** and **d**), and tumors (**e** and **f**), plotted against average absorbed dose (Gy) and effective half-life (hours) for the two methods

The percentage differences in absorbed dose and effective half-life between the 4-point mono-exponential fits and the “original method” are shown in Bland-Altman plots (Fig. 4). The mean percent differences in estimated absorbed dose (Table 1) indicated that absorbed dose is on average slightly overestimated when using the 4 point exponential fit. However, mean differences were low for normal organs and only slightly higher (4.6%) for tumors. Mean differences in effective half-life were also low (< 3%) (Table 2). However, both over- and under-estimates of absorbed dose and effective half-life occurred, as reflected in the 95% intervals; individual differences in absorbed dose were greater than 10% for 8 tumors, ranging up to 57%, and differences in effective half-life were greater than 10% for at least one tumor or normal organ in 23 of the 30 patients, ranging up to 94%, and exceeding 20% for at least one tumor or organ in 9 patients.

**Table 1:** Percentage differences in absorbed dose from “original method” (combination trapezoidal/3-point mono-exponential fit)

Comparison of estimated absorbed dose		Number	Mean % difference from “original method”	95% confidence interval for mean % difference	2.5–97.5 percentile interval	Number of organs/tumors with absolute difference > 10%
4-point mono-exponential fit 0–∞	Tumors	85	4.6%	2.7–6.5%	− 12.8–22.2%	8
	Kidneys	59	3.2%	2.8–3.6%	0.5–5.9%	0
	Liver	30	2.0%	1.1–2.8%	− 2.5–6.4%	0
	Spleen	27	2.8%	2.0–3.5%	− 1.0–6.6%	1
3-point (2, 3, 4) mono-exponential fit 0–∞	Tumors	85	0.8%	0.4–1.3%	− 2.8–4.5%	0
	Kidneys	59	2.4%	1.8–3.1%	− 2.3–7.2%	0
	Liver	30	1.5%	0.9–2.1%	− 1.6–4.6%	0
	Spleen	27	1.5%	0.9–2.2%	− 1.6–4.7%	0
2-point (2, 4) mono-exponential fit 0–∞	Tumors	85	2.6%	1.6–3.5%	− 5.1–10.2%	7
	Kidneys	59	3.4%	2.5–4.2%	− 3.0–9.7%	1
	Liver	30	2.7%	0.5–4.8%	− 8.8–14.1%	3
	Spleen	27	2.2%	0.9–3.5%	− 4.4–8.7%	0
2-point (2, 3) mono-exponential fit 0–∞	Tumors	85	-0.4%	− 3.1–2.4%	− 18.5–17.7%	20
	Kidneys	59	1.9%	0.6–3.3%	− 8.3–12.2%	4
	Liver	30	2.1%	− 3.1–7.4%	− 25.6–29.9%	7
	Spleen	27	1.0%	− 1.9–3.9%	− 13.4–15.4%	3
2-point (3, 4) mono-exponential fit 0–∞	Tumors	85	7.7%	− 0.6–16.0%	− 67.1–82.6%	23
	Kidneys	59	1.5%	1.2–4.3%	− 18.8–21.8%	21
	Liver	30	8.5%	− 0.8–17.7%	− 40.2–57.2%	5
	Spleen	27	3.0%	− 0.4–6.4%	− 14.0–20.0%	3

**Table 2:** Percentage differences in effective half-life from “original method” (combination trapezoidal/3-point mono-exponential fit)

Comparison of estimated half-life		*n*	Mean % difference from “original method”	95% confidence interval for mean % difference	2.5–97.5 percentile interval	% VOIs with absolute % difference > 10%
4-point mono-exponential fit 0–∞	Tumors	85	2.1%	− 2.1 − 6.3%	− 36.0 − 40.2%	28
	Kidneys	59	0.5%	− 1.4 − 2.3%	− 12.8 − 7.6%	7
	Liver	30	− 2.9%	− 5.5 − 0.2%	− 17.1 − 11.4%	5
	Spleen	27	− 0.9%	− 4.7 − 3.0%	− 20.4 − 18.7%	5
3-point (2, 3, 4) mono-exponential fit 0–∞	Tumors	85	*	*	*	*
	Kidneys	59	*	*	*	*
	Liver	30	*	*	*	*
	Spleen	27	*	*	*	*
2-point (2, 4) mono-exponential fit 0–∞	Tumors	85	0.7%	0.2 − 1.3%	− 3.9 − 5.4%	1
	Kidneys	59	0.2%	0.1 − 0.6%	− 2.1 − 1.4%	0
	Liver	30	0.5%	0.2 − 1.3%	− 3.2 − 4.3%	0
	Spleen	27	0.1%	− 0.5 − 0.7%	− 2.9 − 3.1%	0
2-point (2, 3) mono-exponential fit 0–∞	Tumors	85	− 2.7%	− 6.4 − 1.0%	− 35.5 − 30.1%	33
	Kidneys	59	− 1.7%	− 3.8 − 0.4%	− 17.0 − 5.8%	12
	Liver	30	0.9%	− 8.8 − 10.6%	− 49.3 − 51.1%	14
	Spleen	27	− 1.3%	− 5.8 − 3.2%	− 23.2 − 20.7%	9
2-point (3, 4) mono-exponential fit 0–∞	Tumors	85	31.3%	10.3 − 52.3%	− 154.3 − 216.9%	53
	Kidneys	59	10.6%	0.7 − 20.6%	− 62.2 − 46.3%	29
	Liver	30	27.3%	0.9 − 53.7%	− 109.4 − 164.0%	24
	Spleen	27	7.8%	− 1.0 − 16.6%	− 35.3 − 50.9%	17

*Note that the 3-point (2, 3, 4) mono-exponential fit 0–∞ is identical to the fit used for the “original method” combined trapezoidal/mono-exponential fit method: hence, half-life is identical

Visual examination of the observed data points together with the corresponding fitted curves, together with the values of the corresponding *R*^2^, made it clear that the “original method” was a better match to the observed data than 4-time point mono-exponential fit from 0 to infinity. Errors in estimates of absorbed dose and effective half-life in the 4-point mono-exponential fit occurred most notably where initial uptake continued to increase after the early time point. In these cases, the discrepancy in estimated dose was not due to overestimating the integral in the first 24 h, but to the skewed fit resulting from inclusion of the early time point yielding a longer half-life so that the tail end of the fitted curve is higher than its true time course. This comparison demonstrates the risks of including an early time point in the mono-exponential fit. While use of a 4-point single exponential including a 4-h time point would give a fairly accurate absorbed dose for the majority of patient organs, and most tumors too; our findings suggest that it would give rise to a serious error in the absorbed dose for a minority of curves. Moreover, while discrepancy in estimated absorbed dose was only small for most tumors and organs, discrepancies in effective half-life for washout were more frequent and were greater than 10% in at least one tumor or organ in as many as 23 of the 30 patients.

### Comparison of 3 point exponential fit integration from 0 to infinity vs “original method”

Discrepancies between absorbed dose based on the 3-point (24 and 96 h and 1-week post-treatment) mono-exponential fit versus our “original method” were all less than 5% for both tumors and normal organs. The mono-exponential fit in this case is identical to the exponential section of the “original method,” so estimates of effective half-life are unchanged. The small discrepancies in absorbed dose due to the assumption of a mono-exponential curve from injection time included overestimates of absorbed dose in tumors or organs where activity concentration was initially slow to peak, and underestimates in the presence of an initial fast decaying exponential. But these differences were all small (Table 1), indicating that while using trapezoidal integration over the first 24 h may follow the true shape of the curve more closely than the mono-exponential fit; the difference in the total integral is small when considering resulting dose estimates.

### Comparison of 2 point exponential fit integration from 0 to infinity vs “original method”

Calculating absorbed dose and effective half-life based on pairs of scans (scans 2 and 4, scans 2 and 3, scans 3 and 4) yielded results that were highly dependent on which scans were used. Differences from our “original method” in estimates of absorbed dose and effective half-life are summarized in Tables 1 and 2, respectively. In brief, while a 3-point mono-exponential fit is generally influenced most strongly by the first and last points, with relatively little effect of the middle time point, we were still surprised to find the differences in both absorbed dose and effective half-life to be consistently small when comparing mono-exponential fit of scans 2, 3, and 4 with the fit using only scans 2 and 4. On the other hand, mono-exponential fit to pairs of scans 2 and 3 or 3 and 4 gave rise to numerous larger differences in both absorbed dose and effective half-life, as reflected in very large 95 percent intervals, particularly when only scans 3 and 4 were used. These comparisons lead us to believe that dosimetry based on only scans 2 and 4 (24 h and 1-week post-therapy) could give accurate estimates of absorbed dose and effective half-life, whereas dosimetry based on only 2 scans at other pairs of time points could not necessarily be relied on. The 4-h time point was not included in these comparisons, since we already observed (see above) that it was often not representative of the mono-exponential fit out to infinity.

## Discussion

In this retrospective study, we utilized time activity concentration curves formed from 4 serial scans acquired for dosimetry purposes to investigate how best to obtain accurate absorbed dose estimates from these scans, or better still, from an even smaller number of optimally timed post treatment scans. Scan acquisition is demanding in terms of resources and requires patient cooperation.

Estimates of absorbed dose and effective half-life reported in this study are comparable to those reported by others [4, 5, 13, 18, 19]. In all but one of the 30 patients included in this study, absorbed dose to tumor was much higher than absorbed dose to normal organs, confirming high absorbed dose to tumors with relative sparing of normal tissue, as intended. Our calculations showed that higher tumor absorbed dose is due both to higher initial activity concentration (apparent on post-injection images) and longer effective half-life for activity in the tumor versus normal organs.

PRRT with ^177^Lu-DOTATATE is generally repeated at intervals of 5–12 weeks. For personalized dosimetry, a set of 3–4 post-therapy images may be acquired following the first treatment. It may be assumed that absorbed dose for the first treatment is a close approximation for absorbed dose on subsequent treatments. Alternatively, the approximation of constant effective half-life [11, 20] may be used. This makes it possible to obtain more accurate absorbed dose estimates for repeat treatments using a single scan 24-h post-treatment together with effective half-life from first treatment. When using this assumption, inaccuracy in effective half-life has implications for dosimetry for future treatments.

TACCs followed mono-exponential washout overall, but in some cases, the TACCs showed faster washout in the first hours, as reported by others [17], and in other cases an initial rise in activity concentration before subsequent mono-exponential decreases. To the best of our knowledge, this phenomenon of ongoing initial uptake has not been reported elsewhere, and its significance is unknown. Our data did not include sufficient time points to provide exact estimates of timing of peak activity, but approximate interpolation indicated that the peak could occur at least 8–9 h post injection.

In view of the observed diversity, we used combined trapezoidal/mono-exponential fit to estimate the integral under the curve. We did not attempt to fit the model of bi-exponential decrease, because there were TACCs for which this model was clearly unsuitable as described above. In addition, we were concerned that 4 data points would not be sufficient to yield an accurate fit of the larger number of parameters in this model. Further concerns relating to accuracy of the fit are that the exponential fits used relied on linear fit of the logarithms of the TACCs. Future work will include weighted fits as well as estimates of uncertainty of the absorbed dose and half-life and their implications for optimizing absorbed dose calculations for PRRT.

In our method comparisons, we first compared the two methods utilizing all 4 time points, our “original method” and the 4-point mono-exponential fit. Since we found that inclusion of the early (4 h) time point could skew the exponential fit, in our attempts to estimate absorbed dose from only 3 scans, we omitted the 4 h scan, and based absorbed dose estimates on the integral of the resulting 3-point mono-exponential fit extrapolated from injection time to infinity, ignoring divergence from a mono-exponential in the first 24 h for some organs and tumors. This resulted in minimal differences in absorbed dose and effective half-life. Since the mean percentage of the total integral due to the activity concentration in the first 24 h post-injection was relatively low (mean < 23%), as found also by others [21], it is reasonable that even quite large differences in contribution to absorbed dose from activity in this time period would only lead to small differences in total absorbed dose.

Investigating the possibility of basing dosimetry calculations on scans at only two time points, omission of the third (4–5 day) time point, while not as accurate as using 3 time points, resulted in fairly small differences in absorbed dose estimates and effective half-lives. A mono-exponential based on only 2 time points is entirely defined by the 2 points, a simple calculation with no test of goodness of fit, so with no possibility of checking the parameters subsequently used to calculate absorbed dose (from the area under the curve) and effective half-life (from the exponent). The potential impact of inaccuracy of the parameters on absorbed dose and effective half-life remains to be studied. It must also be remembered that organ dosimetry is based on definition of organ and tumor VOIs. When using 3–4 time point curves, sometimes low *R*^2^ alerted us to error in human or automated VOI definition. Without such an indicator, appropriate VOI definition is even more crucial.

Thus, the results of this study suggest that the difference of estimated absorbed dose would not be large by cutting back from 4 to 3 post-treatment scans, omitting the 4 h scan, and that it might even be possible to rely on only 2 scans, 24 h and 7 days after treatment. Further work is required to confirm these results on a larger patient sample.

Others have addressed the question of minimum number and optimal timing of post-treatment scans required for dosimetry. Delker et al. [17] recommended that for kidney dosimetry after ^177^Lu-DOTATATE PRRT, measurements to be included in a mono-exponential fit should be acquired more than 3–5 h after injection. Our findings are in agreement on this point and extend to tumors and additional normal organs. In addition, our findings suggest that it may be advisable to wait longer than 3–5 h. While the MIRD recommendations [14] include imaging at a later time point, methods have been suggested that avoid this. Hanscheid et al. [7] propose that due to the relatively restricted range of effective half-life encountered, accurate dosimetry may be based on only one post-treatment scan 4 days after treatment. Our finding of considerable inter-patient diversity in effective half-life casts doubt on whether this assumption can be extended to all organs and tumors. Others [8–11] have also studied options of estimating absorbed dose from PRRT based on only one scan, generally based on the assumption that there is little deviation from a population average time activity curve. This appears in discordance with the diversity in both absorbed dose and effective half-life observed in the 30 subjects in this study. In future work, a comparison to the methods proposed by these authors could be interesting. Our observation of difference in organ absorbed dose and effective half-life when the 1 week post-treatment scan is omitted also appears to contradict the recommendation that it is sufficient to scan only up to 3 days after PRRT [4, 11] for accurate dosimetry.

Future work is planned, before dispensing with the first and third post-treatment scans, to investigate other information in the 4-point TACCs, including whether the shape of the TACC in the first 24 h might be associated with specific tumor or disease characteristics, and/or implications for tumor response or toxicity in normal organs.

## Conclusion

For personalized dosimetry in radionuclide therapy, dosimetry must be both accurate and accessible [22]. This study indicates that for ^177^Lu-DOTATATE PRRT, good estimates of absorbed dose and effective half-life for washout for organs and tumors may be estimated from scans at 24-h, 72-h and 1-week post-treatment without an earlier scan. It may even be possible to cut back to 2 post-treatment scans, at 24 h and 1 week. However, methods based on only 2 post-treatment scans would be even more user-dependent and require careful scrutiny of the VOIs. Further work on a larger number of patients is required to confirm these conclusions, and to check that other information would not be lost in reducing number of scans.

## Data Availability

The datasets used and/or analyzed during the current study are available from the corresponding author on reasonable request.
